# Treatment of Tislelizumab-Induced Toxic Epidermal Necrolysis and Agranulocytosis: A Case Report and Literature Review

**DOI:** 10.2174/0115748863297885240604111018

**Published:** 2024-06-20

**Authors:** Yanshi Zhou, Honghao Xue, Chenghua Lu, Yemin Zhang, Qingyuan Wu, Jun Zhang, Shiyun Xie, Xiangqian Xu, Xiaoyan Guo

**Affiliations:** 1 Department of Pulmonology, Longhua Hospital, Shanghai University of Traditional Chinese Medicine, Shanghai 200032, China

**Keywords:** Tislelizumab, lung squamous cell carcinoma, toxic epidermal necrolysis, agranulocytosis, case report, immune-related adverse events, immunotherapy

## Abstract

**Background:**

Non-small Cell Lung Cancer (NSCLC) makes up about 85% of lung cancer cases, mainly adenocarcinoma and squamous cell carcinoma. Recently, PD-1 inhibitors have become crucial in NSCLC treatment, significantly enhancing survival for some. However, side effects, like skin reactions and hematotoxicity, limit their use, with drug-induced TEN and immunotherapy-induced agranulocytosis as severe adverse effects.

**Case Presentation:**

Herein, we have reported the case of a 75-year-old male diagnosed with metastatic Lung Squamous cell Carcinoma (LUSC) in the left lung. He received first-line treatment with one cycle of tislelizumab in combination with nab-paclitaxel and carboplatin, after which he developed Toxic Epidermal Necrolysis (TEN) and granulocytopenia. To address these two serious immune-related Adverse Events (irAEs), the patient was administered methylprednisolone in combination with gamma globulin for TEN and dexamethasone in combination with G-CSF for agranulocytosis. Antibiotics were also administered according to the patient’s medication regimen. After treatment, the patient recovered and was discharged from the hospital. It was also noted that the lung tumor condition improved.

**Conclusion:**

Effective management of severe immune-related side effects from tislelizumab, including TEN and agranulocytosis, can be partly achieved through steroids, gamma globulin, G-CSF, and antibiotics. This strategy not only alleviates these adverse effects, but also potentially improves tumor conditions, highlighting the crucial role of vigilant monitoring and management in immunotherapy.

## 1 INTRODUCTION

Tislelizumab, a humanized monoclonal antibody against PD-1, was launched in China in 2019, and has been approved for the treatment of various advanced solid tumors, including Non-small Cell Lung Cancer (NSCLC). Compared to other PD-1 inhibitors, tislelizumab does not bind to the type II macrophage Fcy receptor [1, 2]. This effectively prevents macrophages from attacking T cells, reducing the likelihood of T cell exhaustion and resistance to anti-PD-1 therapy. Nevertheless, administration of tislelizumab could also cause immune-related Adverse Events (irAEs) due to immune upregulation, of which dermatologic toxicity has been reported as the most frequent manifestation [3]. Toxic Epidermal Necrolysis (TEN) is a rare, potentially life-threatening cutaneous adverse reaction characterized by erythema, desquamation, and possible mucosal involvement. Agranulocytosis, alternatively referred to as agranulocytosis or neutropenia, is a serious and acute medical condition characterized by a severe deficiency of neutrophils, which poses significant health risks. Herein, we have presented the first reported co-occurrence of TEN and agranulocytosis in a patient with advanced Lung Squamous cell Carcinoma (LUSC) following treatment with tislelizumab in combination with chemotherapy. After various symptomatic treatments, the patient's symptoms significantly improved.

## 2 CASE PRESENTATION

**Fig. (1):**
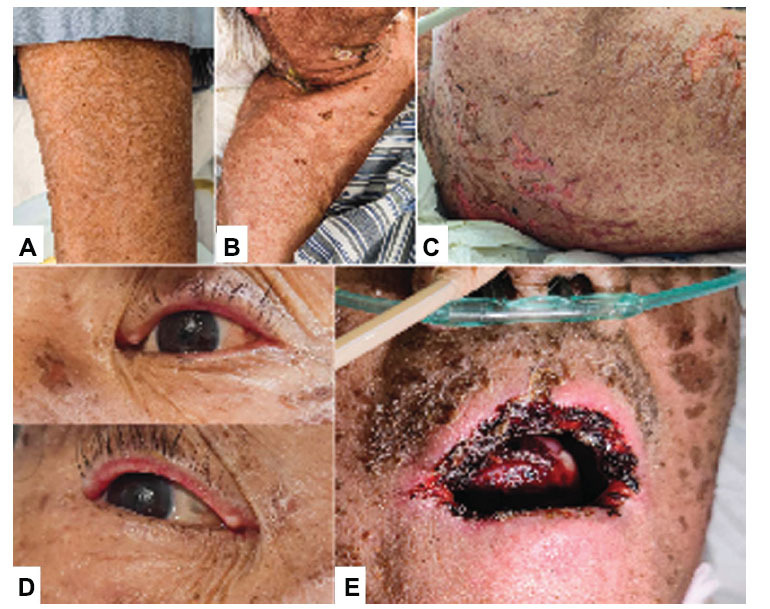
Fever, rash, and ocular involvement in the patient. (**A, B and C**) Skin involvement throughout the body. (**D**) Corneal involvement. (**E**) Involvement of the oral mucosa.

A 75-year-old male with a 50-year history of smoking presented with sputum streaked with blood, followed by a CT scan of the lungs, which revealed a 6 x 4.5 cm mass at the left hilum. A biopsy *via* bronchoscopy confirmed the diagnosis of Lung Squamous cell Carcinoma (LUSC). Further PET-CT imaging showed that the tumor had metastasized to the lower lobe of the left lung and invaded the adjacent left pleura, the right chest wall muscles, and the sixth thoracic vertebra. Genetic testing for the targeted therapies yielded negative results using a 23-gene Next-Generation Sequencing (NGS) panel (Yunying, Jiaxing, China). Treatment was initiated with nab-paclitaxel 400 mg D1, carboplatin 400 mg D1, tislelizumab 200 mg D1, and bone-protective ibandronate. Three days after the treatment, the patient developed a persistent skin rash accompanied by itching and was unresponsive to anti-allergic drugs. On the 14th day, the patient experienced worsening skin lesions, shortness of breath, and scrotal mucosal ulcers. Upon admission, he presented with a high fever, widespread rash, water blisters, and a positive Nikolsky's sign (Fig. **1A-C**). The treatment involved methylprednisolone, gamma globulin, and broad-spectrum antibiotics, leading to skin improvement. On the fourth day, he developed blurred vision and corneal pseudomembranes (Fig. **1D**). Subsequently, painful oral mucosal ulcers emerged, necessitating local care and pain relief (Fig. **1E**).

**Fig. (2):**
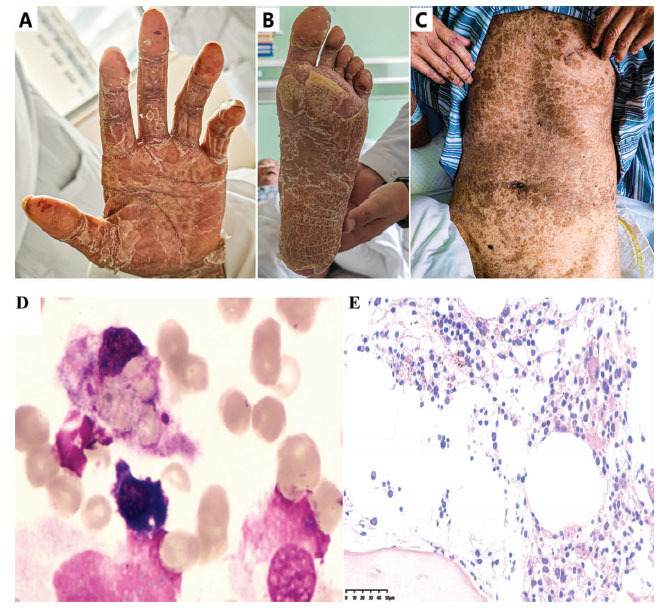
Skin peeling all over the body, active bone marrow proliferation, and granulocyte maturation disorder. (**A,** **B** and **C**) Skin peeling all over the body. (**D**) Bone marrow aspiration smear: active bone marrow proliferation, no primitive/immature cells, and low myeloid proliferation. (**E**) Bone marrow biopsy + immunohistochemistry: the number of granulocytes is small, which can be seen at all stages, and the number is small in the partial mature stage, accompanied by maturation disorders.

Local anti-infection, pain relief, and promotion of epidermal growth were administered as treatments. On the 13th day of hospitalization, the skin began to improve, followed by widespread skin desquamation on the 30th day. Collaboration between dermatologists and ophthalmologists was maintained throughout the study, with a focus on skincare and nutritional support (Fig. **2 A-D**).

**Fig. (3):**
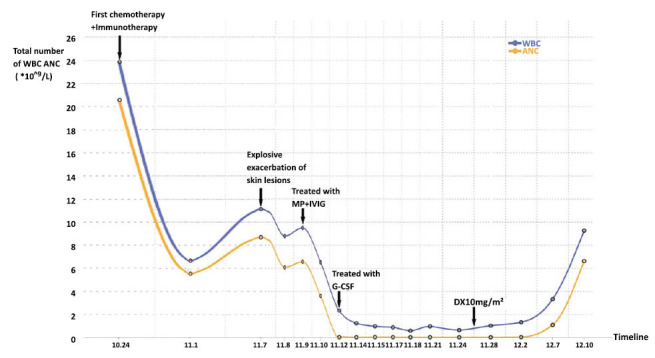
The change curve of white blood cells and neutrophils in the whole course of the disease.

Another serious complication that arose during the patient's hospitalization was neutropenia, which manifested on the fourth day after admission. Routine blood tests revealed a rapid decline in the patient's neutrophil count, which remained critically low at 0.01×10^^^9/L for the subsequent 20 days, accompanied by persistent high fever. To address this, the patient received intravenous G-CSF at a daily dosage of 150ug, along with active anti-infective treatment. Various diagnostic techniques have identified multiple infections, revealing *Escherichia coli* through skin wound cultures, Methicillin-resistant *Staphylococcus aureus* (MRSA) *via* sputum PCR, and herpes virus, hemolytic Staphylococcus, *Rhodococcus erythropolis*, and *Pseudomonas fluorescens* through blood metagenomics NGS. Given the patient's granulocyte deficiency and extensive steroid therapy, infections by *Aspergillus* and *Sporozoans* were also suspected. Consequently, a comprehensive treatment regimen comprising tigecycline, meropenem, and caspofungin was initiated to address these diverse infections. A bone marrow biopsy performed on the 13th day indicated low myeloid cell hyperplasia, the absence of granulocytes at the mesogranule stage, and 1% hemophagocytosis. Immunofluorescence analysis revealed a T lymphocyte prevalence of 69.5%, with a CD4/CD8 ratio of 0.88. Based on these findings, hemophagocytic syndrome was considered, prompting the initiation of dexamethasone induction therapy at a dose of 10 mg/m^2^. G-CSF at 150ug/day continued to support hematopoiesis. Normothermia was achieved on the second day of induction therapy, and by the seventh day, there was an upward trend in neutrophil and leukocyte counts. On December 9th, PET-CT reexamination revealed a reduction in the size and metabolic activity of the primary lesion, with the disappearance of metastatic lesions. The patient ultimately recovered, and was discharged from the hospital (Fig. **3**).

## 3 DISCUSSION

LUSC is the second most common subtype of NSCLC after adenocarcinoma. It is notable for its heterogeneity and high mutational burden, making it difficult to pinpoint and target key mutations [4]. However, in recent years, several immunotherapy-based first-line regimens (with or without chemotherapy) have entered the treatment landscape of LUSC [5-8]. The phase III RATIONALE 307 study led to the approval of tislelizumab with paclitaxel and carboplatin for advanced or metastatic LUSC, noting that the most common severe adverse events of atezolizumab with chemotherapy are hematological, like neutropenia [8]. Multicenter retrospective studies and literature reviews indicate that the incidence of neutropenia in cancer patients treated with Immune Checkpoint Inhibitors (ICIs) ranges from approximately 0.4% to 3.3% [9]. The occurrence rate of granulocytopenia induced by ICIs is estimated to be between 0.013% and 0.14% [10]. The median duration of neutropenia is 13 days, although there is significant variability among the individuals.

Following the administration of tislelizumab combined with chemotherapy, the patient presented a rapid decrease in neutrophil count. Bone marrow biopsy, flow cytometry, and molecular pathology tests have indicated neutropenia to be caused by granulocyte maturation disorder. The bone marrow features of the patient were consistent with previous reports of granulocytopenia caused by ICIs, characterized by granulocytic maturation arrest and interstitial infiltration of T lymphocytes dominated by CD8^+^ T cells [11,12]. Therefore, it was considered that the patient's granulocytopenia was caused by tislelizumab. A meta-analysis has categorized ICI-induced immune-related Neutropenia (irN) into three types: central (25% of cases) marked by T cell-mediated bone marrow aplasia with a poor prognosis, peripheral (12% of cases) associated with anti-neutrophil antibodies and bone marrow hyperplasia with better outcomes, and modified peripheral (63% of cases) showing favorable outcomes but not fitting into the first two categories [12]. The patient's bone marrow results indicated the central type, exhibiting an unfavorable prognosis.

Additionally, the patient developed severe skin reactions after starting tislelizumab. Previous meta-analyses have found that dermatologic toxicities from ICIs occur in 34-42% [13] or 30-68% [14] of cases, with severe reactions, like Stevens-Johnson Syndrome (SJS) or TEN, being rare (less than 1%). In a retrospective study of 20 randomized controlled trials involving 11, 597 patients, ICIs were found to be associated with a significantly increased risk of SJS/TEN, with a median onset time of TEN at 32 days, and among 305 followed-up patients, 69 died due to TEN [15]. In the diagnosis process of TEN for this patient, both drug hypersensitivity syndrome and Acute Generalized Exanthematous Pustulosis (AGEP) were excluded. The diagnosis was confirmed through monitoring the levels of eosinophils and total Immunoglobulin E (IgE), observing the presence of Nikolsky's sign, involvement of multiple mucous membranes, delayed onset of symptoms, and the absence of pus in the blisters, culminating in the diagnosis with widespread epidermal detachment.

The patient experienced TEN and agranulocytosis. Reports have indicated around 13% of SJS/TEN patients to experience reduced neutrophils [16]. Experimental studies have highlighted a unique NET-necroptosis axis in SJS/TEN, triggering CD8^+^ T cells and leading to neutrophil depletion through NETosis [17]. This suggests that the decrease in neutrophil count in SJS/TEN results from peripheral consumption. In this case, persistent agranulocytosis, attributed to bone marrow maturation disorder, was compounded by skin disorders contributing to the reduction in peripheral granulocytes, explaining the ongoing agranulocytosis in the patient. Throughout the treatment, CSFs were administered long-term, chiefly for managing neutropenia. Additionally, prior studies have suggested that CSFs promote rapid epithelial cell regeneration by increasing neutrophil counts and mobilizing hematopoietic stem cells to aid in cell proliferation [18].

## CONCLUSION

A 75-year-old male patient with LUSC experienced severe immune-related side effects, including TEN and agranulocytosis, from tislelizumab, nab-paclitaxel, and carboplatin treatment. Effective management using steroids, gamma globulin, G-CSF, and antibiotics led to the resolution of these side effects and tumor improvement, emphasizing the need for careful monitoring and management in immunotherapy.

## Data Availability

Not applicable.

## LIST OF ABBREVIATIONS

NSCLC: Non-Small Cell Lung Cancer
LUSC: Lung Squamous Cell Carcinoma
irAEs: Immune-Related Adverse Events
TEN: Toxic Epidermal Necrolysis
ICIs: Immune Checkpoint Inhibitors
irN: Immune-Related Neutropenia
SJS: Stevens-Johnson Syndrome

## STANDARDS OF REPORTING

CARE guidelines were followed.
